# Assessment of Lubiprostone as an Adjunct Therapy for Bowel Preparation in Colonoscopy: A Meta‐Analysis of Randomized Controlled Trials

**DOI:** 10.1002/jgh3.70186

**Published:** 2025-05-15

**Authors:** Fariha Hasan, Muhammad Shahzil, Ayesha Liaquat, Fatima Farooqi, Avneet Singh, Alexander Garcia, Muhammad Yafaa Naveed Chaudhary, Dushyant Singh Dahiya, Tanay‐Veer Gandhi, Andrew Alabd, Rachel Frank

**Affiliations:** ^1^ Department of Internal Medicine Cooper University Hospital Camden New Jersey USA; ^2^ Penn State Health Milton S. Hershey Medical Centre Hershey Pennsylvania USA; ^3^ Department of Internal Medicine Dow University of Health Sciences Karachi Pakistan; ^4^ Department of Internal Medicine Indiana University Southwest New Albany Indiana USA; ^5^ Gastroenterology & Hepatology The University of Kansas School of Medicine Kansas City Kansas USA; ^6^ Cooper Medical School of Rowan University Camden New Jersey USA; ^7^ Department of Gastroenterology Cooper University Hospital Camden New Jersey USA

**Keywords:** bowel preparation, colon cancer diagnostics, colon cancer prevention, colon cancer screening, colon cancer therapeutics, colonoscopy, lubiprostone, pharmacotherapeutics

## Abstract

**Introduction:**

The quality of bowel preparation has a significant impact on the success of colonoscopy. Currently, osmotically balanced polyethylene glycol electrolyte (PEG‐E) solutions are most commonly used for bowel preparation. Recently, lubiprostone (LBP) has been considered a potentially effective adjunct to PEG. We conducted a meta‐analysis of randomized controlled trials (RCTs) to evaluate the safety and efficacy of LBP in bowel preparation for colonoscopy.

**Methods:**

Following PRISMA guidelines, we systematically screened PubMed, Embase, Web of Science, and Cochrane Library for RCTs on LBP as an adjunct to PEG‐E for improving bowel preparation quality for colonoscopy. Statistical analysis was performed on RevMan, using a random‐effects model with the generic inverse variance method to address clinical heterogeneity; results were significant at *p* < 0.05. Outcomes were reported as relative risks and standard errors.

**Results:**

This meta‐analysis included seven RCTs with 1206 patients. Adding LBP did not increase the likelihood of an excellent bowel preparation [RR = 1.28, 95% CI: 0.94–1.74, *p* = 0.12] or contribute to poor preparation [RR = 0.61, 95% CI: 0.36–1.04; *p* = 0.07]. It also did not affect procedure time [MD = −0.74, 95% CI: −2.91–1.43; *p* = 0.50], polyp detection rate [RR = 1.07, 95% CI: 0.90–1.26; *p* = 0.45], or adenoma detection rate [RR = 1.09, 95% CI: 0.75–1.57; *p* = 0.66].

**Conclusion:**

Our meta‐analysis found that LBP, explored as an adjunct to PEG‐E solutions for bowel preparation, offers no significant additive effect on preparation quality before colonoscopy.

## 1 Introduction

Colonoscopy is the standard procedure for screening and monitoring colorectal cancer (CRC) as it allows for the visualization of the entire colon [1]. Approximately 17 million colonoscopies are performed annually in the United States (US) [2]. The efficacy of colonoscopy relies heavily on adequate bowel preparation, which is essential for optimal mucosal visualization and the detection of lesions [1]. Inadequate bowel preparation impedes the detection of smaller lesions and increases colonoscopy time, thereby increasing the likelihood of procedure‐related adverse events and patient dissatisfaction [3, 4]. Additionally, insufficient bowel preparation necessitates repeat examinations and a higher frequency of surveillance tests, contributing to a substantial cost burden [5].

Currently, the most commonly used laxative for bowel preparation is the osmotically balanced polyethylene glycol‐electrolyte (PEG‐E) bowel lavage solution. However, the large volume of fluid (approximately 4 L) is not generally well tolerated, leading to nausea, vomiting, and abdominal cramps [6]. Consequently, split‐dose regimens and adjunct therapies to PEG‐E have been sought to improve tolerance and bowel preparation quality [7].

One emerging adjunct therapy is lubiprostone (LBP), which has been shown to improve the quality of bowel preparation [8]. LBP is a selective type 2 chloride channel activator that increases fluid volume in the intestine, softens stool, and shortens intestinal transit time. It was approved by the US Food and Drug Administration in 2007 for treating chronic idiopathic constipation and irritable bowel syndrome with constipation (IBS‐C) [9, 10]. While its side effects include nausea and vomiting, it is generally well tolerated and boasts an excellent safety profile [11, 12].

Existing evidence evaluating LBP's additive effect on bowel preparation quality before colonoscopy has been inconsistent and inconclusive. Some studies have reported it to be as effective as PEG‐E therapy alone [13], while others have found it to be more effective [14, 15]. In addition, a previously conducted meta‐analysis by Peng et al. [16] had insufficient evidence, thereby reducing the statistical power of the analysis. Given the newly available data, we conducted a comprehensive literature search and meta‐analysis of randomized controlled trials (RCTs) to assess the safety and efficacy of LBP in improving bowel preparation among patients undergoing colonoscopy.

## 2 Materials and Methods

### 2.1 Data Sources and Search Strategy

This meta‐analysis is performed in accordance with the Preferred Reporting Items for Systematic Review and Meta‐Analysis (PRISMA) guidelines for updated meta‐analysis [17] and follows the structure laid out by the Cochrane Collaboration [18]. Two independent reviewers (LA and SM) conducted a comprehensive electronic search of PubMed, Embase, Web of Science, and Cochrane CENTRAL from their inception to April 2024 using an extensive search strategy that involved all possible terms and abbreviations of key terms “LBP,” “colonoscopy,” “bowel preparation,” “bowel cleansing,” “colon preparation,” and “colon cleansing” along with MeSH terms and Boolean operators “AND” and “OR”. In addition, the bibliographic sections of the selected articles and reviews were manually screened for any other relevant studies. The search strategy is available in Table S1.

### 2.2 Study Selection and Data Extraction

The eligibility criteria for our meta‐analysis included all RCTs assessing the safety and efficacy of LBP as an adjunct therapy to PEG for bowel preparation before colonoscopy. No language restriction was applied. The primary outcome was bowel preparation efficacy, defined as excellent or poor preparation, as well as the Boston Bowel Preparation Score (BBPS). The secondary outcomes included Ottawa Bowel Preparation Score (OBPS), length of colonoscopy, adenoma detection rate, polyp detection rate, number of adverse events, types of adverse events (nausea, vomiting, dizziness, abdominal bloating, and cramps), withdrawal time, patient compliance, and satisfaction.

### 2.3 Data Screening and Extraction

The patient demographics and outcomes data from the finalized studies were independently assessed and extracted by two reviewers (LA and SM). Any disagreements between the two reviewers were resolved through discussion and mutual consensus with the senior investigator (HF).

### 2.4 Risk‐Of‐Bias and Certainty‐Of‐Evidence Assessment

Since all the included studies were RCTs, the risk of bias was assessed using the Cochrane Risk of Bias 2 tool [19], which is based on the following five criteria: randomization process, allocation concealment, blinding of participants and personnel, blinding of outcome assessment, incomplete outcome data, selective reporting, and other biases. Two reviewers (FF and SM) independently applied the risk assessment tools to all included studies and classified the risk of bias of each study as low risk, high risk, or some concerns. Any differences in determining the risk of bias or justification were resolved through discussion and mutual consensus with the senior investigator (HF). The Grading of Recommendations Assessment, Development, and Evaluation (GRADE) approach was then used to rate the certainty of the evidence for each outcome of interest, using a minimally contextualized approach with a threshold of any difference between groups, based on the following criteria: risk of bias, inconsistency, imprecision, indirectness, and other considerations [20].

### 2.5 Statistical Analysis

A meta‐analysis was performed on RevMan (version 5.4; Copenhagen: The Nordic Cochrane Centre, The Cochrane Collaboration) using the Mantel–Haenszel random‐effects model with generic inverse variance to account for clinical heterogeneity. The heterogeneity in the effect sizes was reported using Higgin's *I* ^2^ statistic, where *I* ^2^ greater than 50% showed significance [21]. The results were reported as risk ratios (RRs) with 95% confidence intervals (CIs) for dichotomous variables and mean differences with 95% confidence intervals (CIs) for continuous variables. A *p*‐value < 0.05 was considered significant in all cases. As per the Cochrane guidelines, a publication bias assessment could not be conducted as fewer than 10 studies were included in the meta‐analysis [22].

## 3 Results

**FIGURE 1:**
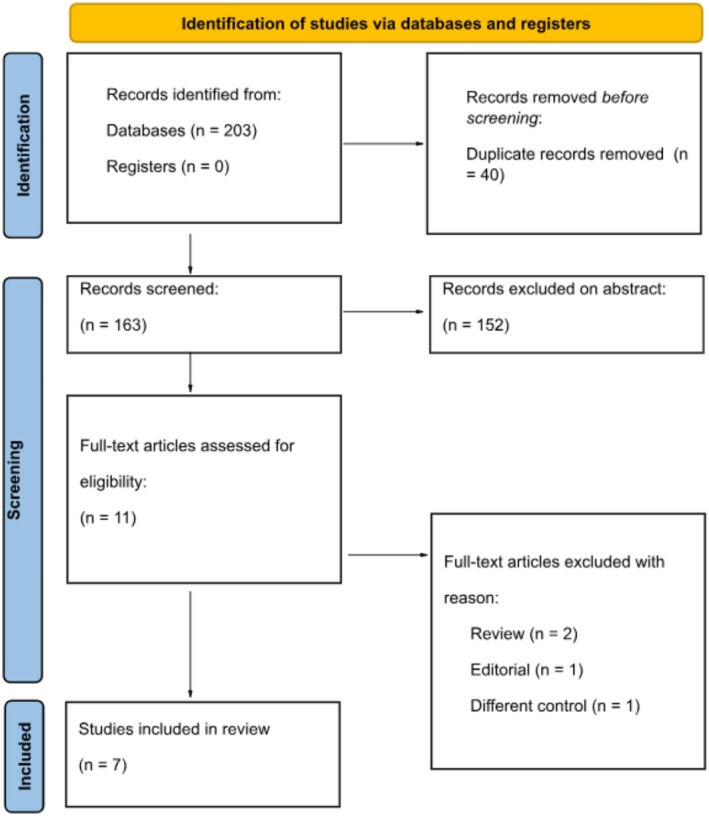
PRISMA flow diagram for included and excluded studies.

The initial literature search yielded 203 results, of which 40 duplicates were identified and removed, resulting in 163 remaining studies. In the end, seven studies were finalized for data extraction after excluding reviews, case reports, letters to the editor, and studies with different controls (Figure 1).

All the included studies involved the use of LBP for bowel preparation before colonoscopy as an intervention [12, 13, 14, 15, 23, 24, 25]. The characteristics of the included studies are presented in Table 1.

**TABLE 1:** Characteristics of included studies.

Study ref.	Country	Study			Bowel preparation regimen	Adjunct regimen		Bowel preparation scale	Sedation	Dietary instructions	Modified Jadad score
		Type	Population	Duration		Lubiprostone	Control				
Stengel and Jones [15]	USA	RCT	Outpatient screening; colonoscopy, ≥ 18 years	2 months	4 L PEG‐E, Split dose	24 mcg of lubiprostone at noon the day before colonoscopy	Placebo	Ottawa bowel preparation scale	Fentanyl or meperidine and midazolam	Standardized diet until 4 pm and subsequently only a clear liquid diet	5
Grigg et al. [14]	USA	RCT	Outpatient screening colonoscopy with AODM; ≥ 50 years	21 months	4 L PEG‐E, Single dose	24 mcg lubiprostone 2 h before PEG‐E and 2 h after PEG‐E	No placebo	Aronchick scale	Fentanyl and midazolam, or diprivan	Only a clear liquid diet	1
Hjelkrem et al. [24]	USA	RCT	Outpatient screening colonoscopy; ≥ 18 years	12 months	255 g PEG without electrolytes mixed with 64 oz. Gatorade, Split dose	24 mcg lubiprostone at noon the day before colonoscopy	No placebo	Ottawa bowel preparation scale	Fentanyl and midazolam	Only a clear liquid diet	2
Sofi et al. [13]	USA	RCT	Screening, surveillance or diagnostic colonoscopy; ≥ 18 years	Not available	4 L PEG‐E, Split dose	24 mcg lubiprostone at two nights before colonoscopy, subsequent 24 mcg lubiprostone at breakfast, lunch, and dinner on the day before colonoscopy	Placebo	Ottawa bowel preparation scale	Not available	Only a clear liquid diet	5
Banerjee et al. [23]	India	RCT	Outpatient colonoscopy; 18–75 years	5 months	2 L PEG‐E, Single dose	24 mcg lubiprostone 1 h before PEG‐E	Placebo	Boston bowel preparation scale	Propofol	Standardized diet	5
Sirinawasatien et al. [12]	Thailand	RCT	Outpatient elective colonoscopy; 18–75 years	19 months	PEG 4000 plus electrolytes diluted with 2 L plain water	24 mcg lubiprostone 2 h before PEG on pre‐procedure day	No placebo	Boston bowel preparation scale	Not available	Low residue diet	TBD
Tangvoraphonkchai et al. [25]	Thailand	RCT	Colonoscopy and had constipation; 18–75 years	13 months	PEG‐ELS, Split dose	24 mcg lubiprostone at 2 days before colonoscopy, subsequent 24 mcg lubiprostone on the day before colonoscopy	No placebo	Ottawa bowel preparation scale	Not available	Only a clear liquid diet	Not available

Abbreviations: PEG‐E, polyethylene glycol‐electrolyte; RCT, randomized controlled trial; USA: United States of America.

The demographic characteristics and patient profiles from the included studies are summarized as follows: 1206 participants were included in the analysis, with 595 being administered LBP and 611 receiving a placebo. The mean ages were 55.6 ± 21.04 and 55.2 ± 22.78 for the group administered LBP and placebo, respectively. Males comprised 53.4% and 51.1% of each group, respectively. The baseline characteristics of patients from the included studies are presented in Table 2.

**TABLE 2:** Baseline characteristics of patients from included studies.

Study ref.	Sample size		Mean age (years)		Males (fractions) (%)	
	Lubiprostone	Control	Lubiprostone	Control	Lubiprostone	Control
Stengel and Jones [15]	95	96	55.4 ± 5.2	55.9 ± 4.8	46/95 (48.4)	51/96 (53.1)
Grigg et al. [14]	17	24	Not available	Not available	Not available	Not available
Hjelkrem et al. [24]	101	100	55.4 ± 5.7	54.1 ± 5.3	48/101 (47.5)	49/100 (49.0)
Sofi et al. [13]	57	66	56.1 ± 9.4	55.8 ± 9.1	29/57 (50.9)	32/66 (48.5)
Banerjee et al. [23]	221	221	45.9 ± 15.2	45.8 ± 14.7	160/221 (72.4)	154/221 (69.7)
Sirinawasatien et al. [12]	70	70	58.8 ± 10.8	58.5 ± 11.2	44/70 (62.9)	40/70 (57.1)
Tangvoraphonkchai et al. [25]	34	34	61.7 ± 3.10	61.3 ± 13.2	13/34 (38.2)	10/34 (29.4)

### 3.1 Primary Outcomes

#### 3.1.1 Excellent Preparation

**FIGURE 2:**
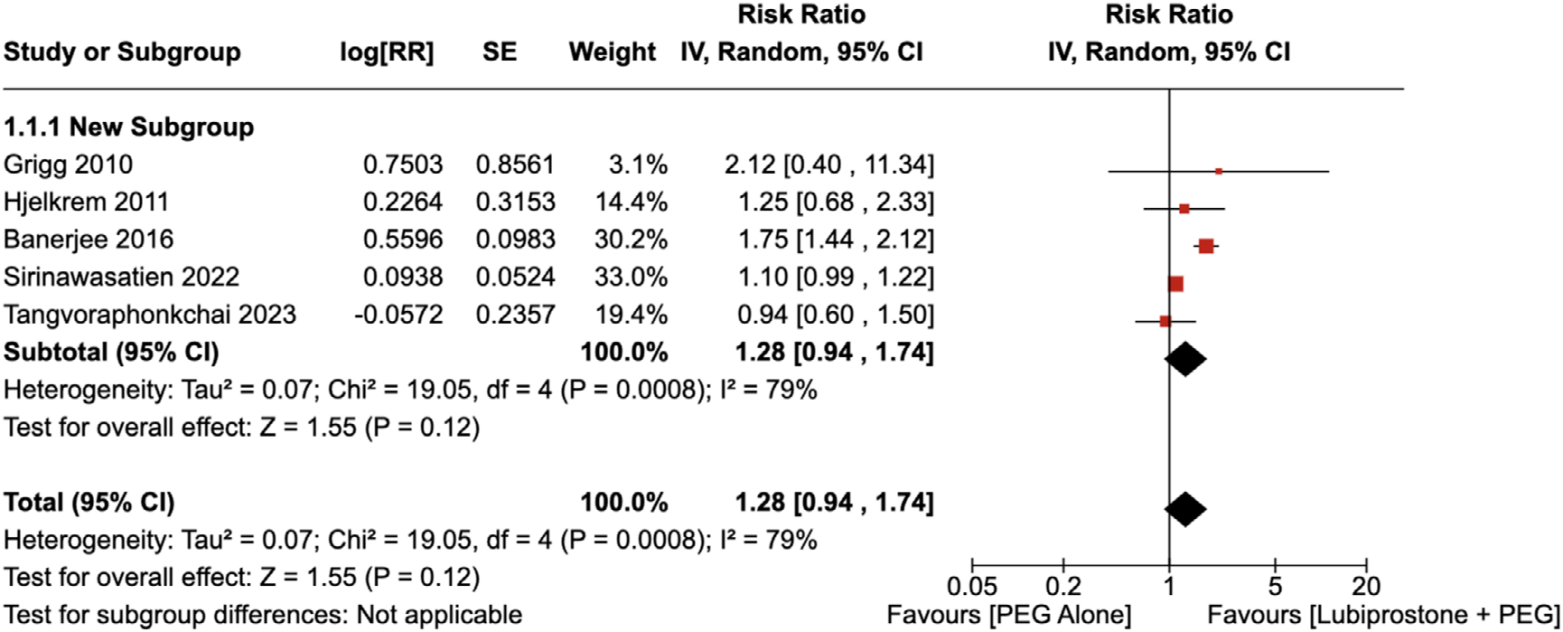
Forest plot showing excellent preparation for colonoscopy observed with the administration of Lubiprostone compared to the use of placebo.

There was no significant improvement in excellent preparation for colonoscopy observed with the administration of LBP compared to the use of placebo [RR = 1.28 (95% CI, 0.94–1.74; *p* = 0.12)], as shown in Figure 2. The heterogeneity was calculated as *I* ^2^ = 79%. Upon conducting a sensitivity analysis by removing Banerjee et al. the heterogeneity dropped to *I* ^2^ = 0%. However, the difference in excellent preparation for colonoscopy remained insignificant between the groups [RR = 1.10 (95% CI, 0.99–1.21), *p* = 0.07] as shown in Figure S1A.

#### 3.1.2 Poor Preparation

**FIGURE 3:**
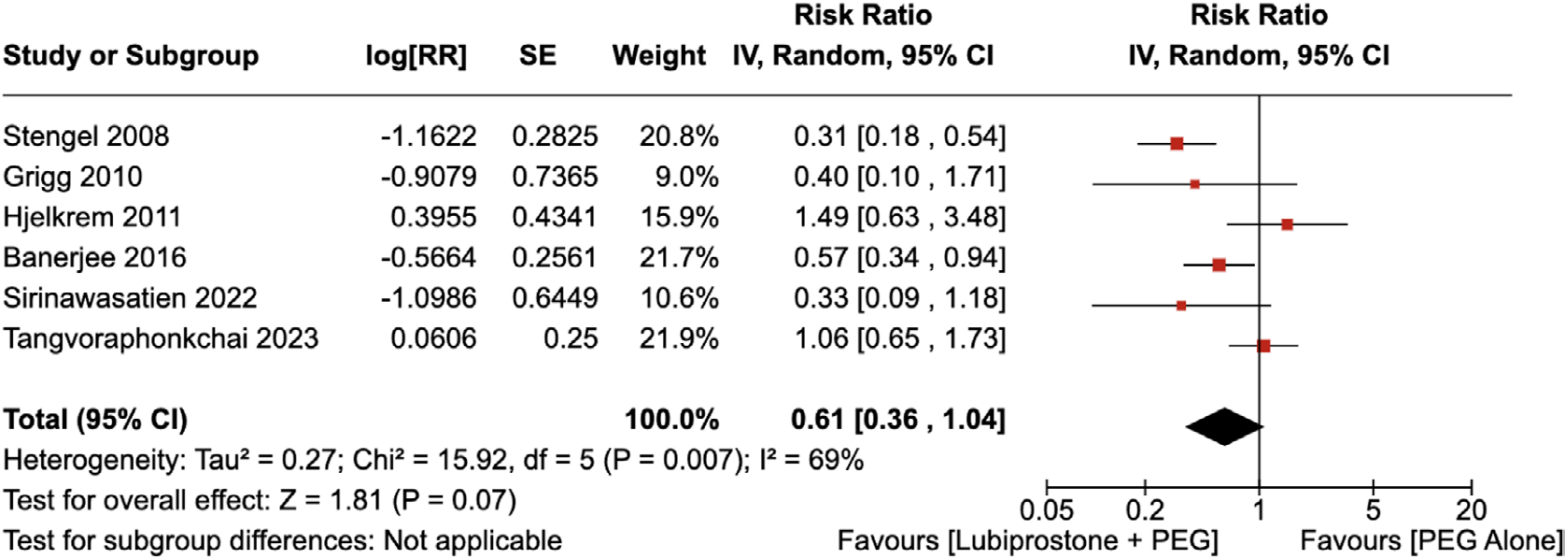
Forest plot showing poor preparation for colonoscopy observed with the administration of Lubiprostone compared to the use of placebo.

There was no significant difference in poor preparation for colonoscopy observed with the administration of LBP compared to the use of placebo [RR = 0.61 (95% CI, 0.36–1.04; *p* = 0.07)], as shown in Figure 3. The heterogeneity was calculated as *I* ^2^ = 69%. Upon conducting a sensitivity analysis by removing the study by Stengel et al. the heterogeneity dropped to *I* ^2^ = 49%. However, no significant difference was observed in poor preparation for colonoscopy between the two groups [RR = 0.75 (95% CI, 0.46–1.22), *p* = 0.25] as shown in Figure S1B.

## 4 Boston Bowel Preparation Score‐Total and Ascending Colon

**FIGURE 4:**
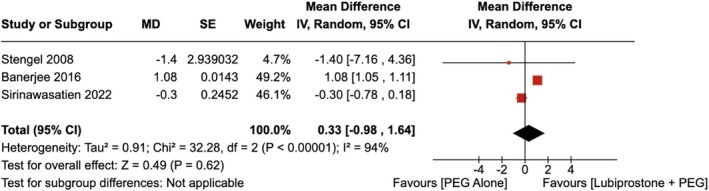
Forest plot showing Total Boston Bowel Preparation Score for colonoscopy observed with the administration of Lubiprostone compared to the use of placebo.

No significant improvement was observed in the total BBPS with the administration of LBP compared to the use of placebo [RR = 0.33 (95% CI, −0.98–1.64 *p* = 0.62)], as shown in Figure 4. The heterogeneity was calculated as *I* ^2^ = 94%, indicating high heterogeneity.

**FIGURE 5:**
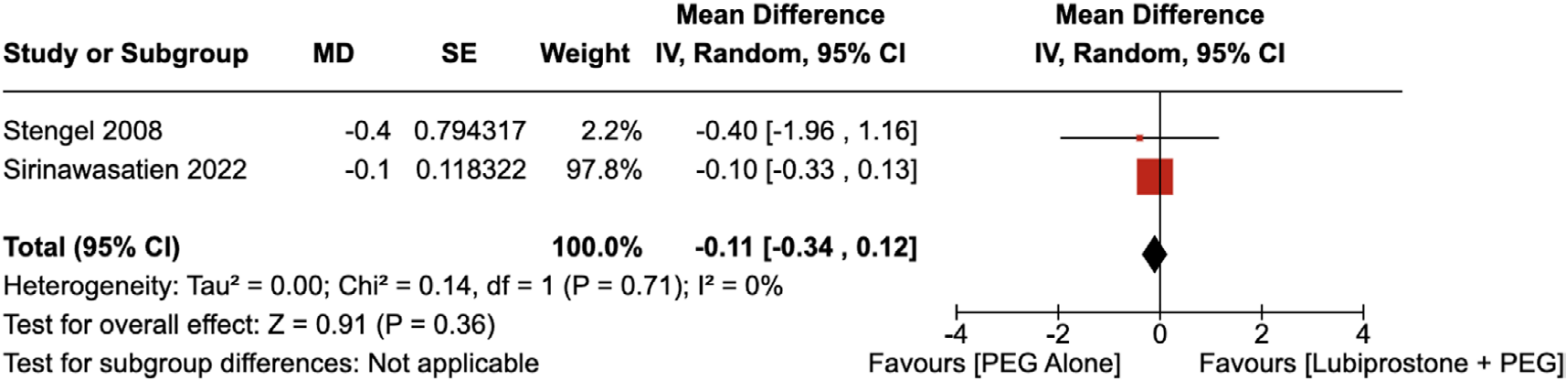
Forest plot showing Boston Bowel Preparation Score of the ascending colon observed with the administration of Lubiprostone compared to the use of placebo.

Similarly, there was no statistically significant improvement in the BBPS of the ascending colon with LBP administration compared to the use of placebo [RR = −0.11 (95% CI, −0.34–0.12; *p* = 0.36)], as shown in Figure 5. The heterogeneity was calculated as *I* ^2^ = 0%, indicating low heterogeneity.

### 4.1 Secondary Outcomes

#### 4.1.1 Ottawa Bowel Preparation Score—Total

**FIGURE 6:**
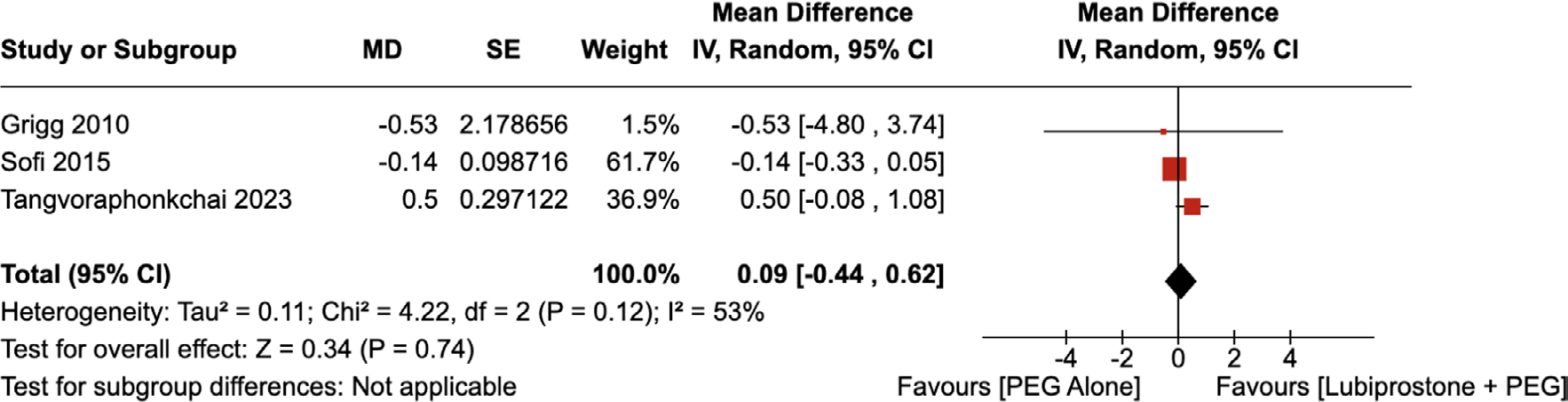
Forest plot showing the total Ottawa Bowel Preparation Score in colonoscopy observed with the administration of Lubiprostone compared to the use of placebo.

There was no significant improvement in the total OBPS observed with the administration of LBP compared to the use of placebo [RR = 0.09 (95% CI, −0.44–0.62; *p* = 0.74)], as shown in Figure 6. The heterogeneity was calculated as *I* ^2^ = 53%, indicating high heterogeneity.

#### 4.1.2 Length of Colonoscopy

**FIGURE 7:**
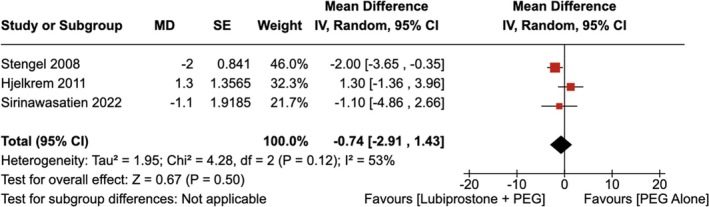
Forest plot showing length of colonoscopy observed with the administration of Lubiprostone compared to the use of placebo.

There was no statistically significant difference in the length of colonoscopy observed with the administration of LBP compared to the use of placebo [RR = −0.74 (95% CI, −2.91–1.43; *p* = 0.50)], as shown in Figure 7. The heterogeneity was calculated as *p* = 0.12; *I* ^2^ = 53%, indicating high heterogeneity. Upon conducting sensitivity analysis by removing Hjelkrem et al. the heterogeneity dropped to *I* ^2^ = 0%. Additionally, pooled analysis showed that the administration of LBP significantly decreased the length of colonoscopy compared to the administration of placebo [RR = −1.85 (95% CI, −3.36–0.35), *p* = 0.02] as shown in Figure S1C.

#### 4.1.3 Cecal Intubation Rate

**FIGURE 8:**
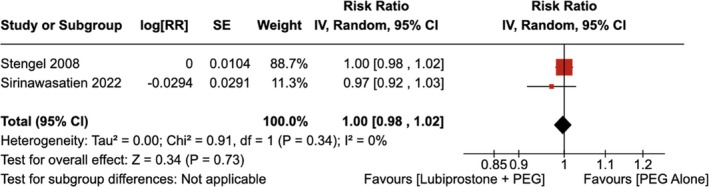
Forest plot showing cecal intubation rate observed with the administration of Lubiprostone compared to the use of placebo.

There was no statistically significant improvement in the cecal intubation rate seen with the administration of LBP compared to the use of placebo [RR = 1.00 (95% CI, 0.98–1.02; *p* = 0.73)], as shown in Figure 8. The heterogeneity was calculated as *I* ^2^ = 0%, indicating low heterogeneity.

#### 4.1.4 Polyp Detection Rate

**FIGURE 9:**
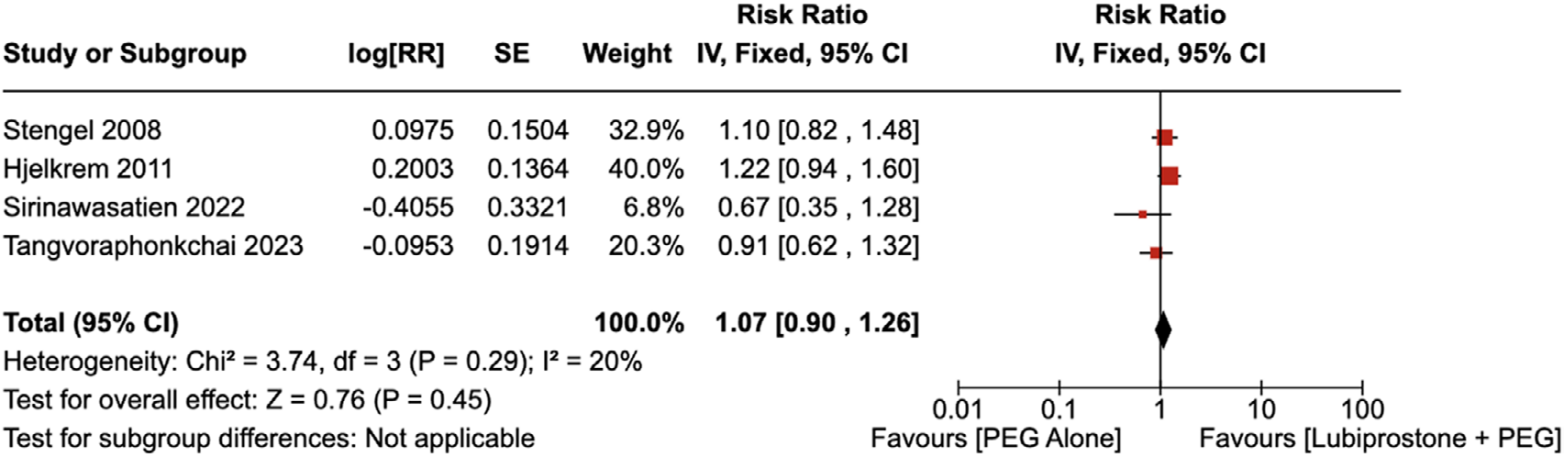
Forest plot showing polyp detection rate observed with the administration of Lubiprostone compared to the use of placebo.

There was no statistically significant increase in the polyp detection rate observed with the administration of LBP compared to the use of placebo [RR = 1.07 (95% CI, 0.90–1.26; *p* = 0.45)], as shown in Figure 9. The heterogeneity was calculated as *I* ^2^ = 20%, indicating low heterogeneity.

#### 4.1.5 Adenoma Detection Rate

**FIGURE 10:**
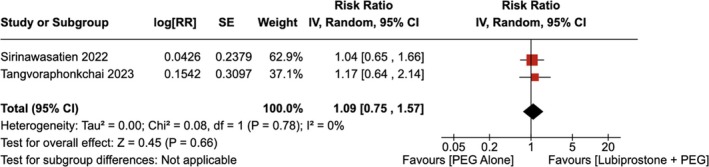
Forest plot showing adenoma detection rate observed with the administration of Lubiprostone compared to the use of placebo.

No statistically significant increase in the adenoma detection rate was observed with the administration of LBP compared to the use of placebo [RR = 1.09 (95% CI, 0.75–1.57; *p* = 0.66)], as shown in Figure 10. The heterogeneity was calculated as *I* ^2^ = 0%, indicating low heterogeneity.

#### 4.1.6 Any Adverse Effects or Complications

**FIGURE 11:**
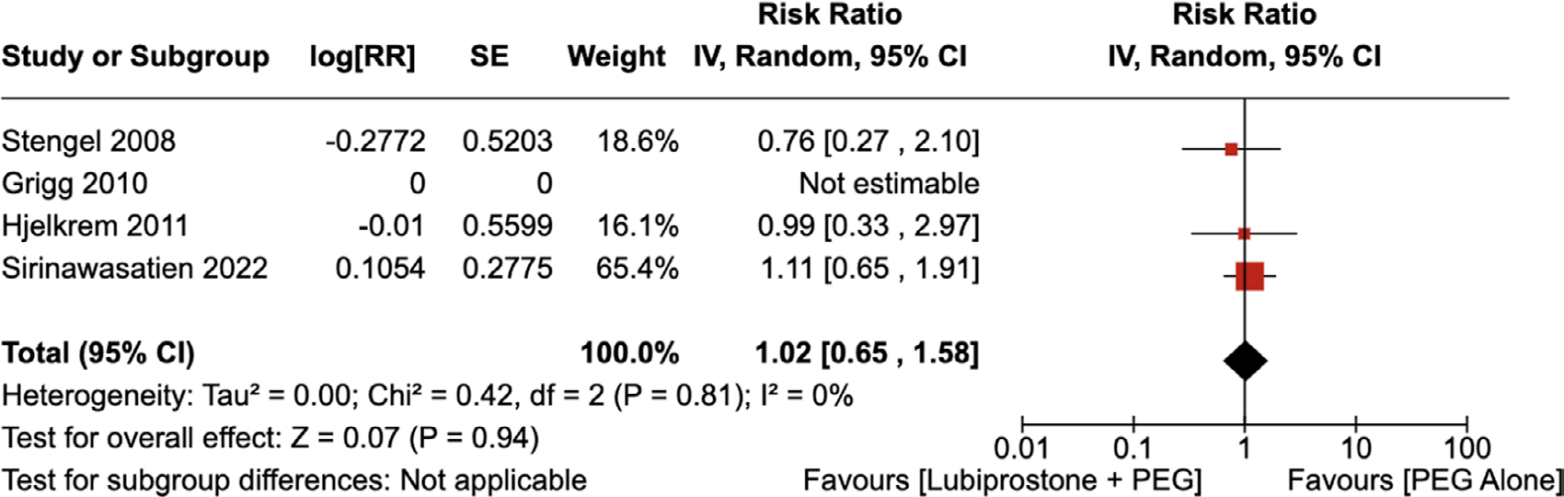
Forest plot showing any adverse effects or complications of colonoscopy observed with the administration of Lubiprostone compared to the use of placebo.

There was no statistically significant change in the total number of adverse effects or complications observed with the administration of LBP compared to the use of placebo [RR = 1.02 (95% CI, 0.65–1.58; *p* = 0.94)], as shown in Figure 11. The heterogeneity was calculated as *I* ^2^ = 0%, indicating low heterogeneity.

#### 4.1.7 Abdominal Cramps/Pain

**FIGURE 12:**
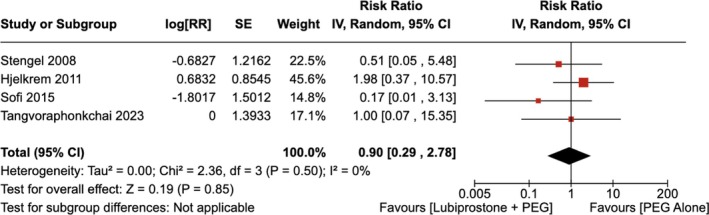
Forest plot showing incidence of abdominal cramps/pain after colonoscopy observed with the administration of Lubiprostone compared to the use of placebo.

There was no statistically significant difference in the number of patients experiencing abdominal pain with the administration of LBP compared to the use of placebo [RR = 0.90 (95% CI, 0.29–2.78; *p* = 0.85)], as shown in Figure 12. The heterogeneity was calculated as *I* ^2^ = 0%, indicating low heterogeneity.

#### 4.1.8 Abdominal Bloating

**FIGURE 13:**
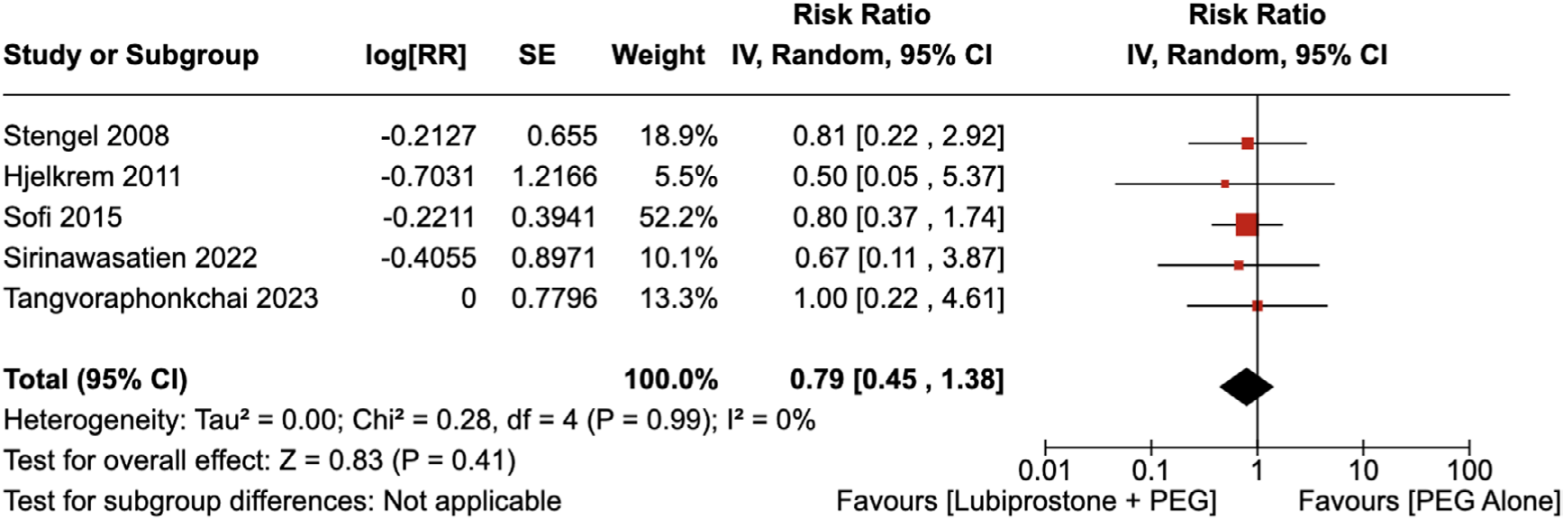
Forest plot showing the incidence of abdominal bloating after colonoscopy observed with the administration of Lubiprostone compared to the use of placebo.

There was no statistically significant difference in the number of patients experiencing abdominal bloating with the administration of LBP compared to the use of placebo [RR = 0.79 (95% CI, 0.45–1.38; *p* = 0.41)], as shown in Figure 13. The heterogeneity was calculated as *I* ^2^ = 0%, indicating low heterogeneity.

### 4.2 Other Outcomes

There was no significant improvement in the incidence of nausea [RR = 1.03 (95% CI, 0.73–1.44; *p* = 0.89)], vomiting [RR = 0.63 (95% CI, 0.08–4.99; *p* = 0.66)], and dizziness [RR = 1.77 (95% CI, 0.54–5.81; *p* = 0.34)] observed with the administration of LBP compared to the use of placebo as seen in Figures S2A–C, respectively. No significant difference was observed in the rate of compliance [RR = 1.01 (95% CI, 0.96–1.07; *p* = 0.74)], satisfaction [RR = 1.00 (95% CI, 0.88–1.13; *p* = 0.99)] or the withdrawal time [RR = −0.20 (95% CI, −2.17–1.78; *p* = 0.85)] between LBP and the control as seen in Figures S2D–F, respectively.

## 5 Risk of Bias Assessment

The risk of bias assessment was applicable in all seven studies. All studies showed a low risk of bias (Table S2).

## 6 GRADE Assessment

The GRADE assessment was made in all the outcome studies. All outcomes showed high certainty (Table S3).

## 7 Discussion

This updated meta‐analysis of RCTs examines the addition of LBP to PEG bowel preparation before colonoscopy. Our findings reveal no significant differences in bowel preparation quality, bowel preparation scores, or pathology detection rates. Secondary outcomes, including parameters influencing the duration of the colonoscopy, the proportion of patients experiencing adverse events, and patients' subjective assessments of compliance and satisfaction, also showed no statistically significant changes. These results contrast with those of the previous meta‐analysis, which, while reporting no difference in procedure time, pathology detection rates, and number of adverse events between the two groups, did highlight significantly improved preparation quality in the LBP group [16].

Adequate bowel preparation is paramount for a successful colonoscopy, significantly impacting safety, diagnostic accuracy, and examination efficiency [1]. Consequently, evaluating bowel preparation quality is essential when assessing any preparation regimen. Our meta‐analysis demonstrates no significant differences in preparation quality, classified as excellent or poor, between the LBP and the standard PEG groups. Similarly, assessments using the Ottawa and Boston Bowel Preparation Scores indicate that there is no benefit from adding LBP to the PEG regimen. These findings are consistent with the majority of individual studies included in the analysis, except for the studies by Banerjee et al. [23] and a previous meta‐analysis [16], which suggested that combining PEG with LBP improved bowel preparation. This improvement was attributed to accelerated colonic transit time and increased frequency of bowel movements [16]. It is essential to consider several limitations of the studies, including the lack of standardization of the bowel preparation scales and significant heterogeneity among the trials included in prior meta‐analyses. Our analysis, however, carefully categorized the outcome data to align with all trials, enabling us to report the findings confidently and address the limitations of previous studies. Although LBP has been effective in treating chronic constipation [26], it has not been proven to be efficacious for bowel preparation when combined with PEG. The consistent results across all bowel preparation parameters confirm the non‐superiority of adding LBP to the PEG regimen over the standard PEG regimen.

Chang et al. identified that suboptimal bowel preparation during the initial colonoscopy and high‐risk adenomas are significant predictors of missed advanced adenomas. Additionally, both the adenoma detection rate and the rate of missed adenomas are associated with interval CRC [27, 28]. LBP, recognized for its laxative effects [11], was hypothesized to enhance bowel preparation regimens. However, our analysis showed no improvement in adenoma or polyp detection rates. Suboptimal bowel preparation has also been found to affect procedural time, thereby impacting both the cecal intubation rate and withdrawal time [27, 28]. In our study, the LBP group showed no significant differences from the PEG group in reducing the cecal intubation rate or withdrawal time, indicating no superiority of the LBP group over the PEG‐only group.

Patient tolerability is a critical concern, as bowel preparation regimens often lead to adverse gastrointestinal symptoms, sleep disturbances, and dietary issues [29]. These challenges can diminish regimen adherence, resulting in poor preparation quality, reduced compliance with screening colonoscopy, and lower post‐procedure satisfaction. Although new regimens aim to alleviate these adverse events, our analysis did not demonstrate any additional benefit from adding LBP to the PEG regimen. This finding aligns with previous reports associating LBP with several gastrointestinal side effects, with nausea being the most prominent, along with other dyspeptic symptoms, including delayed gastric emptying [26, 30]. Therefore, including LBP does not improve the tolerance of bowel preparation regimens.

Our meta‐analysis had certain limitations. Various PEG regimens, including PEG‐E and PEG with Gatorade, were administered in different dosing regimens, such as single and split doses. Additionally, the volume of PEG used varied across regimens, ranging from 2 to 4 L. Differences in dietary instructions also contributed to the observed heterogeneity. Moreover, the study populations varied, encompassing the general population undergoing outpatient elective screening colonoscopy, except for Tangvoraphonkchai et al. which included patients with chronic constipation [25]. This variation in study populations could introduce further heterogeneity. Therefore, the interpretation of results should be approached cautiously considering these limitations.

It is crucial to prioritize advancements that enhance early detection rates of colorectal carcinoma, minimize adverse events, and improve patient compliance and comfort during colonoscopy procedures. A recent network meta‐analysis assessing different bowel preparation regimens demonstrated that incorporating ascorbic acid and simethicone can effectively cleanse the bowel and alleviate abdominal bloating. Additionally, combining PEG with sodium picosulfate/magnesium citrate improved cecal intubation rates and increased patient satisfaction, particularly in the ease of undergoing repeat colonoscopy screenings [31]. Future advancements will be instrumental in identifying superior regimens that improve patient comfort and enhance the diagnostic accuracy of colonoscopy.

## 8 Conclusion

In conclusion, our updated meta‐analysis reveals no significant benefits of adding LBP to PEG regimens for bowel preparation. The data indicate no improvement in bowel preparation quality, pathology detection rates, or patient satisfaction. These findings align with most individual studies and suggest that LBP, despite its efficacy in treating chronic constipation, does not offer superior outcomes in bowel preparation for colonoscopy. Future research should continue to explore optimal regimens to improve CRC screening and patient compliance.

## Conflicts of Interest

The authors declare no conflicts of interest.

## Supporting information


**Data S1.** Supporting Information.

## Data Availability

All data generated or analyzed in this study are included in this published article. Additional inquiries can be addressed to the corresponding author.
