# Supplementary material for: Assessment of Lubiprostone as an Adjunct Therapy for Bowel Preparation in Colonoscopy: A Meta‐Analysis of Randomized Controlled Trials
Source: JGH Open. 2025 May 15;9(5):e70186. doi: 10.1002/jgh3.70186 (PMC12079760; doi:10.1002/jgh3.70186)
Supplement: Supplementary file 1 — Data S1. Supporting Information. [file JGH3-9-e70186-s001.docx]

**Supplementary Material**

Table 1: Search Strategy

| Database | Search Strategy/Keywords | Articles Retrieved |
| --- | --- | --- |
| Pubmed | (Lubiprostone OR RU-0211 OR amitiza) AND (Macrogol OR PEG OR Polyethylene OR Polyethylene Oxide* OR Oxide, Polyethylene OR Oxides, Polyethylene OR Polyethyleneoxide* OR Polyoxyethylene* OR Polyglycol* OR Glycol, Polyethylene OR Glycols, Polyethylene OR Carbowax OR Cathartics OR Bowel Evacuants OR Evacuants, Bowel OR Purgatives OR Bowel Preparation Solutions OR Preparation Solutions, Bowel OR Solutions, Bowel Preparation) AND (Bowel Preparation OR Bowel Cleansing OR Bowel Preparation OR Colonoscopy OR Colon Cleansing OR Colon Preparation) | 14 |
| Embase | (Lubiprostone OR RU-0211 OR amitiza) AND (Macrogol OR PEG OR Polyethylene OR Polyethylene Oxide* OR Oxide, Polyethylene OR Oxides, Polyethylene OR Polyethyleneoxide* OR Polyoxyethylene* OR Polyglycol* OR Glycol, Polyethylene OR Glycols, Polyethylene OR Carbowax OR Cathartics OR Bowel Evacuants OR Evacuants, Bowel OR Purgatives OR Bowel Preparation Solutions OR Preparation Solutions, Bowel OR Solutions, Bowel Preparation) AND (Bowel Preparation OR Bowel Cleansing OR Bowel Preparation OR Colonoscopy OR Colon Cleansing OR Colon Preparation) | 148 |
| Web of Science | (Lubiprostone OR RU-0211 OR amitiza) AND (Macrogol OR PEG OR Polyethylene OR Polyethylene Oxide* OR Oxide, Polyethylene OR Oxides, Polyethylene OR Polyethyleneoxide* OR Polyoxyethylene* OR Polyglycol* OR Glycol, Polyethylene OR Glycols, Polyethylene OR Carbowax OR Cathartics OR Bowel Evacuants OR Evacuants, Bowel OR Purgatives OR Bowel Preparation Solutions OR Preparation Solutions, Bowel OR Solutions, Bowel Preparation) AND (Bowel Preparation OR Bowel Cleansing OR Bowel Preparation OR Colonoscopy OR Colon Cleansing OR Colon Preparation) | 22 |
| Cochrane CENTRAL | (Lubiprostone OR RU-0211 OR amitiza) AND (Macrogol OR PEG OR Polyethylene OR Polyethylene Oxide* OR Oxide, Polyethylene OR Oxides, Polyethylene OR Polyethyleneoxide* OR Polyoxyethylene* OR Polyglycol* OR Glycol, Polyethylene OR Glycols, Polyethylene OR Carbowax OR Cathartics OR Bowel Evacuants OR Evacuants, Bowel OR Purgatives OR Bowel Preparation Solutions OR Preparation Solutions, Bowel OR Solutions, Bowel Preparation) AND (Bowel Preparation OR Bowel Cleansing OR Bowel Preparation OR Colonoscopy OR Colon Cleansing OR Colon Preparation) | 19 |


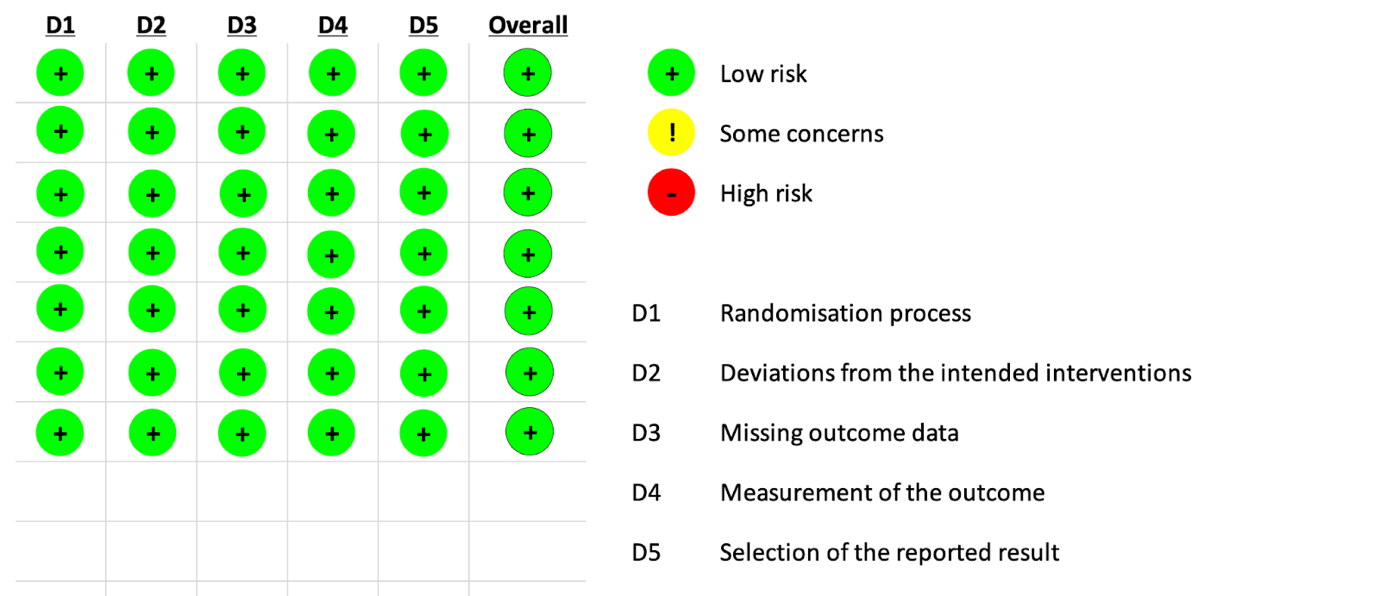


Table 2: Cochrane risk-of-bias (RoB) assessment for randomized-controlled trials


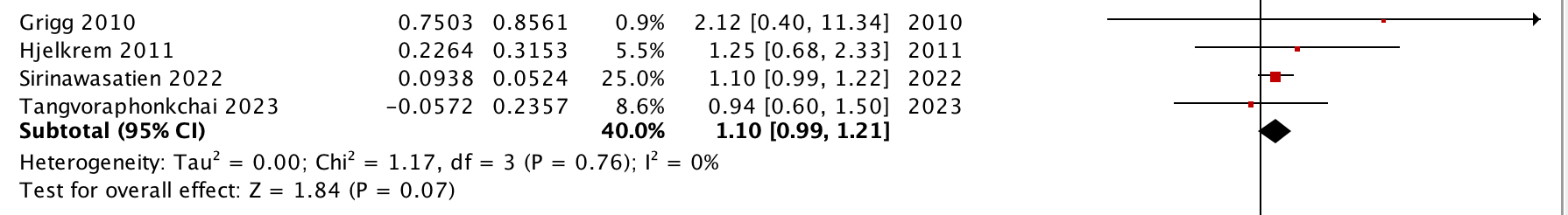


Figure 1A: Forest plot showing excellent preparation for colonoscopy observed with the administration of Lubiprostone compared to the use of placebos after conducting sensitivity analysis.


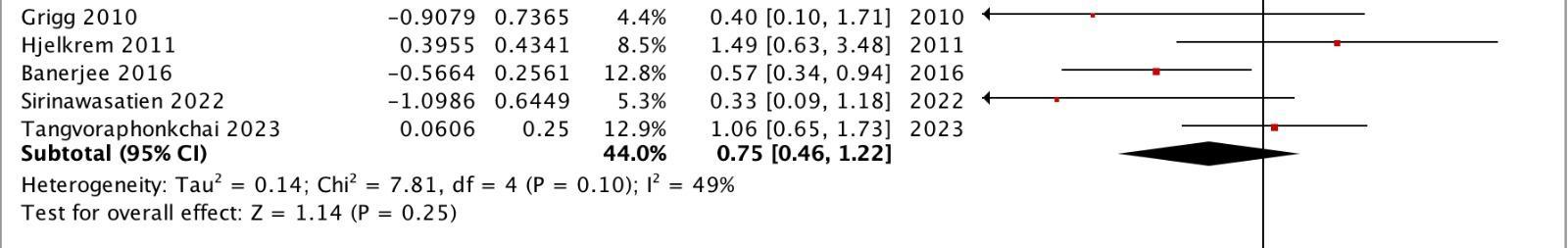


Figure 1B: Forest plot showing poor preparation for colonoscopy observed with the administration of Lubiprostone compared to the use of placebo after conducting sensitivity analysis.

*
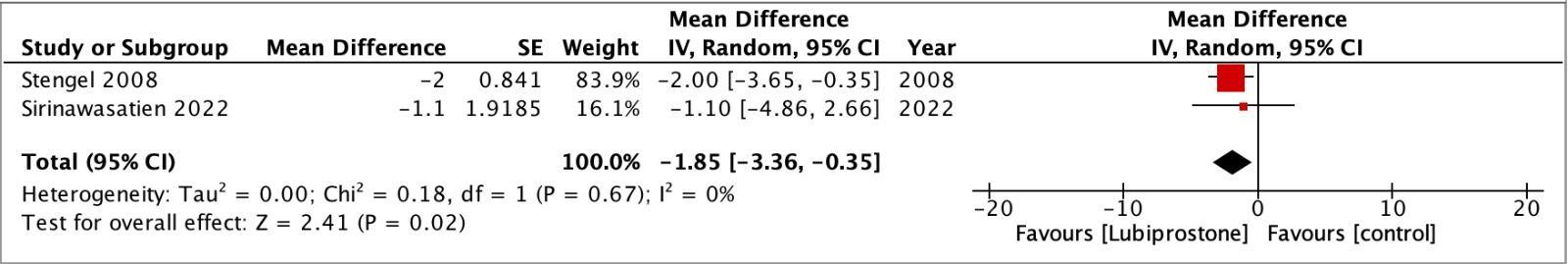
*

Figure 1C: Forest plot showing the length of colonoscopy observed with the administration of Lubiprostone compared to the use of placebo after conducting sensitivity analysis.


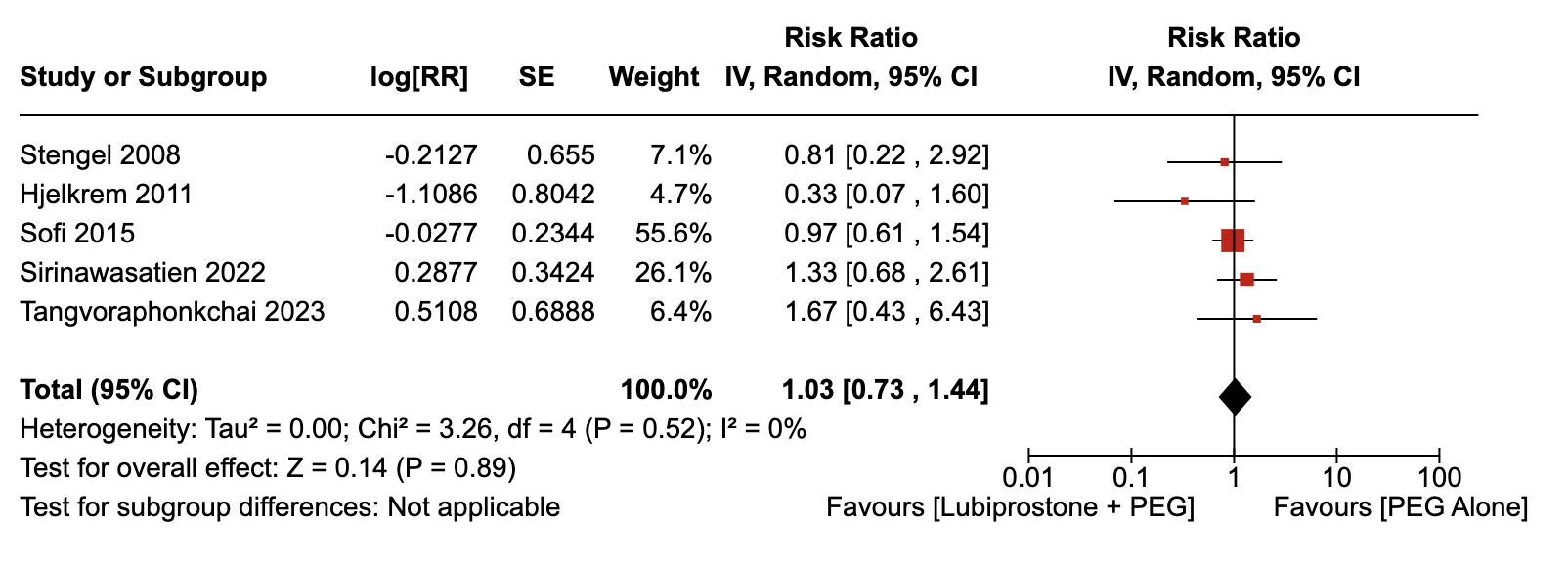

*Figure 2A: Forest plot showing incidence of nausea after colonoscopy observed with the administration of Lubiprostone compared to the use of placebo.*


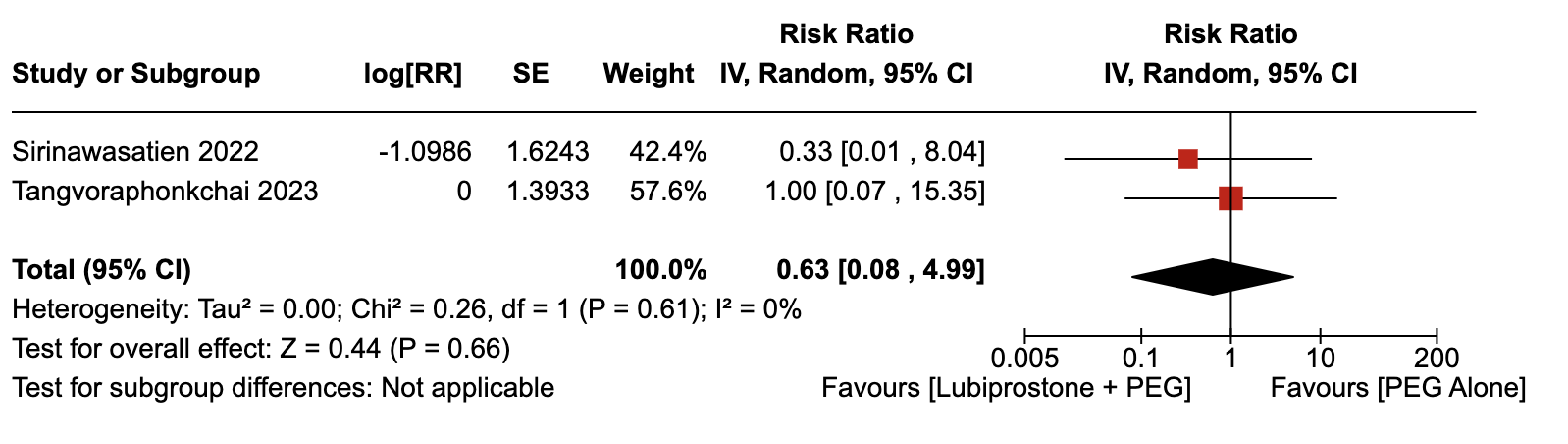


*Figure 2B: Forest plot showing incidence of vomiting after colonoscopy observed with the administration of Lubiprostone compared to the use of placebo.*


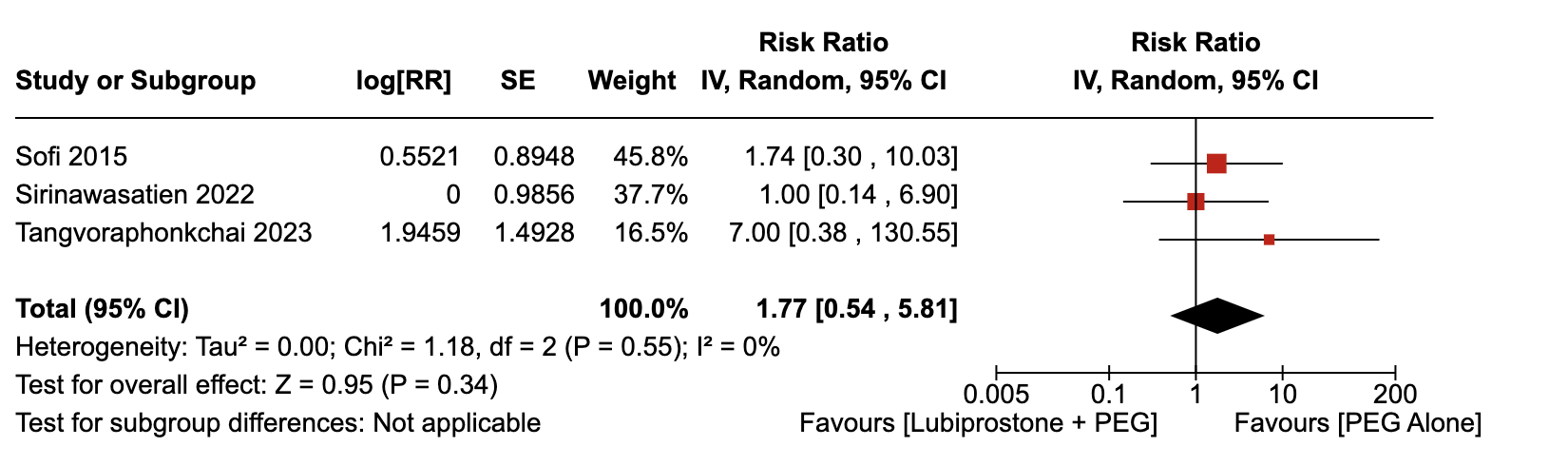


*Figure 2C: Forest plot showing incidence of dizziness after colonoscopy observed with the administration of Lubiprostone compared to the use of placebo.*


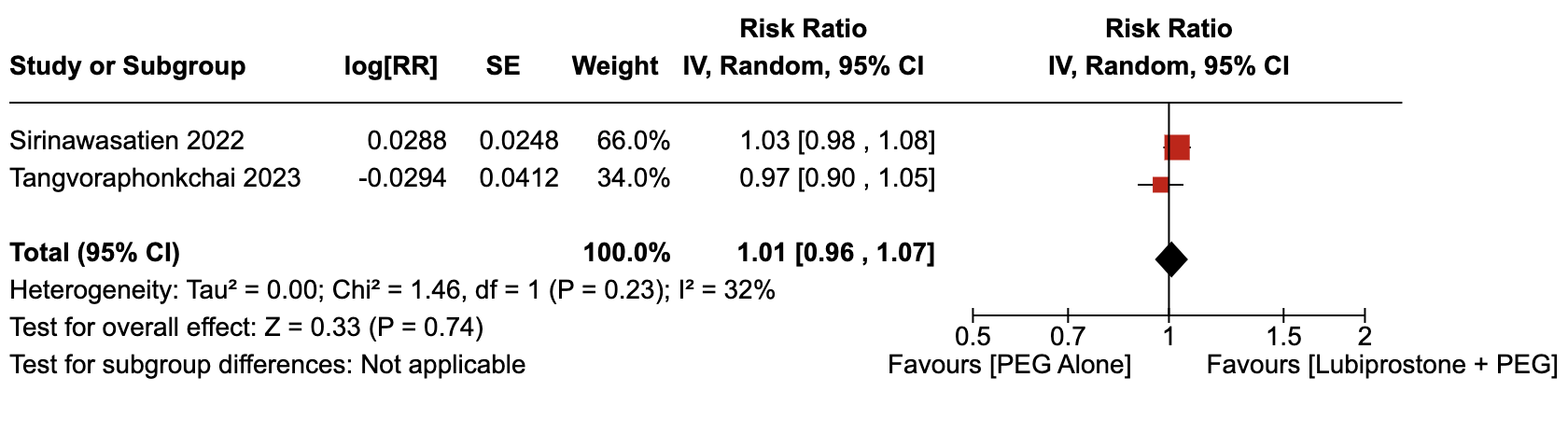


*Figure 2D: Forest plot showing compliance for colonoscopy observed with the administration of Lubiprostone compared to the use of placebo.*


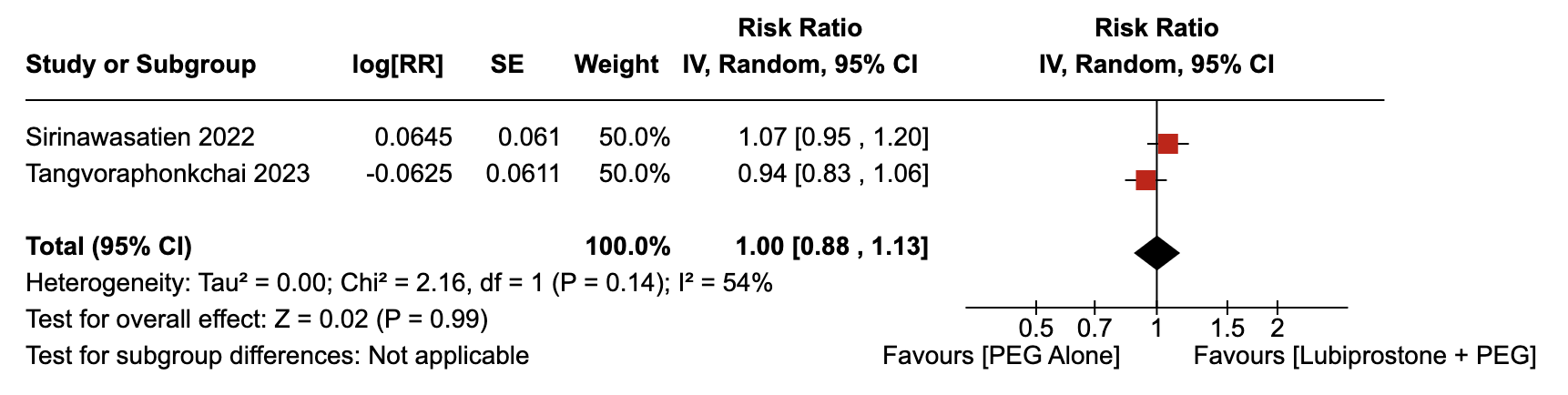


*Figure 2E: Forest plot showing satisfaction after colonoscopy observed with the administration of Lubiprostone compared to the use of placebo*.


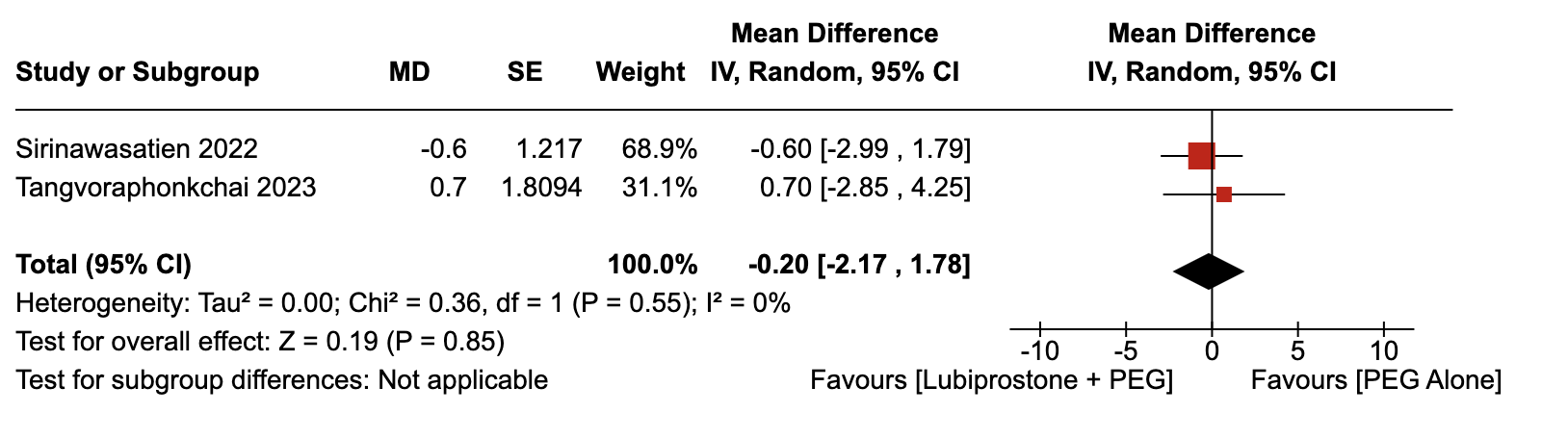


*Figure 2F: Forest plot showing withdrawal time (min) in colonoscopy observed with the administration of Lubiprostone compared to the use of placebo.*


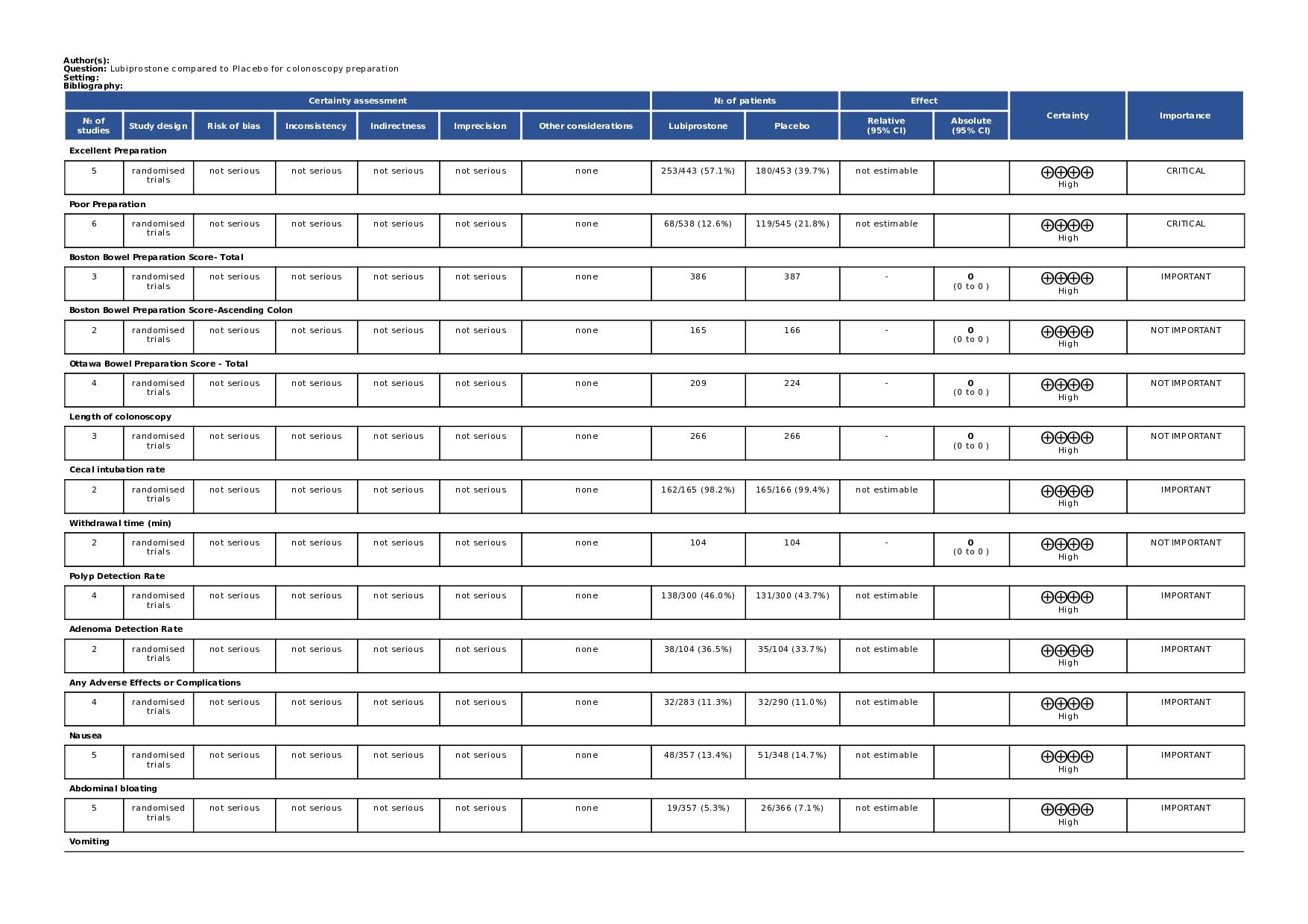

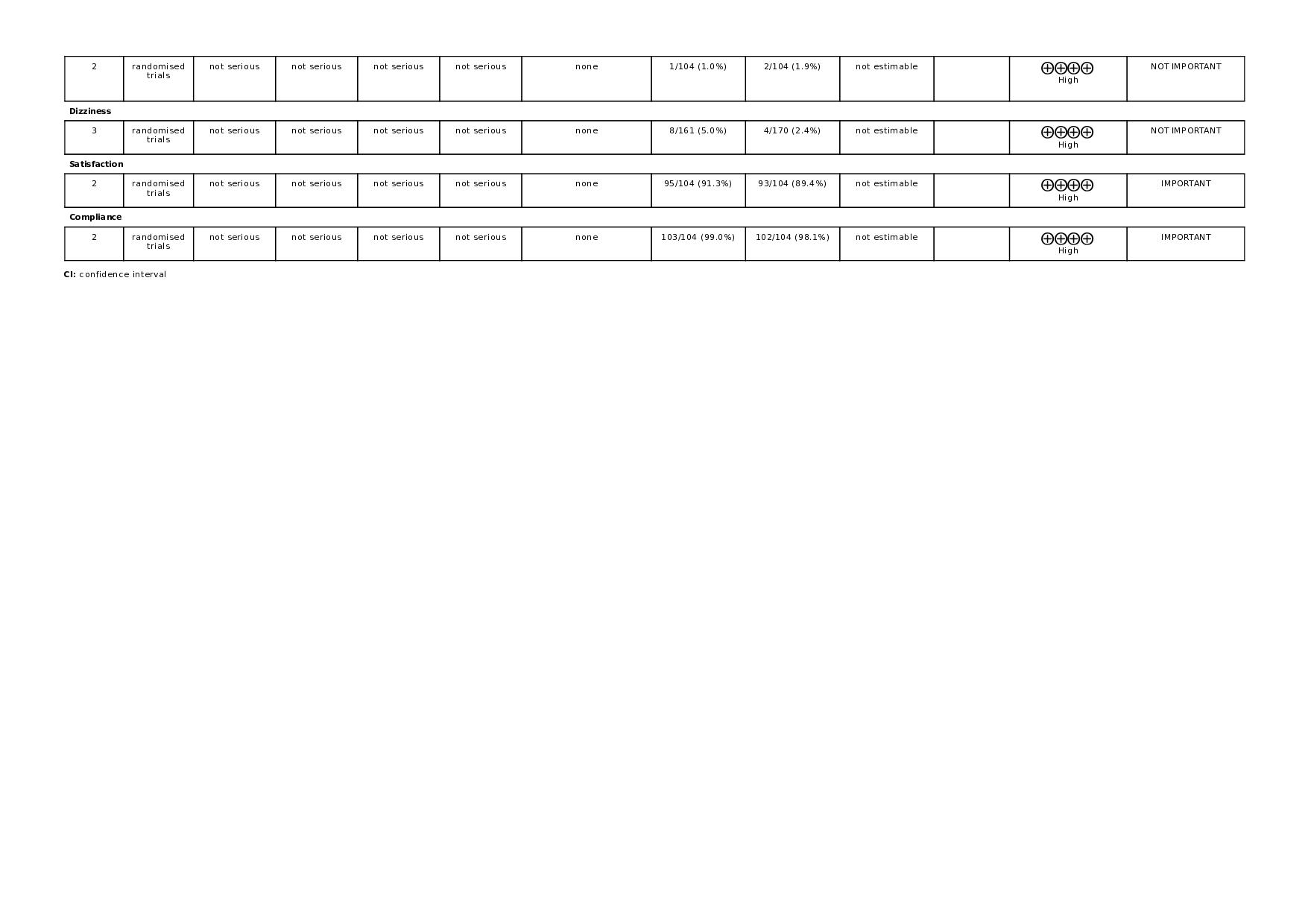


Table 3: Summary of Findings Table using GRADE assessment of outcomes
